# The Urinary Phosphate to Serum Fibroblast Growth Factor 23 Ratio Is a Useful Marker of Atherosclerosis in Early-Stage Chronic Kidney Disease

**DOI:** 10.1371/journal.pone.0160782

**Published:** 2016-08-09

**Authors:** Hodaka Yamada, Makoto Kuro-o, Kazuo Hara, Yuichiro Ueda, Ikuyo Kusaka, Masafumi Kakei, San-e Ishikawa

**Affiliations:** 1 Department of Medicine, Jichi Medical University Saitama Medical Center, 1-847 Amanuma-cho, Omiya-ku, Saitama, 330-8503, Japan; 2 Center for Molecular Medicine, Jichi Medical University, 3311-1 Yakushiji Shimotsuke, Tochigi, 329-0498, Japan; 3 Division of Endocrinology and Metabolism, International University of Health and Welfare Hospital, 537-3 Iguchi, Nasushiobara, Tochigi, 329-2763, Japan; National Center for Scientific Research Demokritos, GREECE

## Abstract

**Background:**

Fibroblast growth factor 23 (FGF23) regulates mineral homeostasis. In developed renal dysfunction, FGF23 levels increase to maintain the phosphate excretion capacity. However, in diabetic patients with early-stage renal impairment, the FGF23 elevation is not very sensitive. We hypothesized that urinary phosphate (U-P)/serum FGF23 ratio would theoretically be an index that reflects the number of nephrons (nephron index). In this study, we determined whether the nephron index would be associated with renal function and vascular diseases in diabetic patients.

**Methods:**

In total, 142 patients with diabetes mellitus were enrolled. The nephron index was calculated using the following formula: U-P (mg/day)/ serum FGF23 (pg/ml).

**Results:**

The mean age was 63 ± 11 years and eGFR levels were 79.5 ± 25.4 ml/min/1.73 m^2^, respectively. Thirty patients had a medical history of macroangiopathy. The Nephron index was significantly decreased in subjects with macroangiopathy compared with those without macroangiopathy. A multivariate analysis of risk factors for macroangiopathy revealed that duration of diabetes, eGFR, and nephron index were significantly associated with a higher frequency of arteriosclerotic disease.

**Conclusion:**

These findings suggest that a decrease in nephron index reflects early-stage renal impairment and is an independent risk factor of macroangiopathy in diabetic patients.

## Introduction

Fibroblast growth factor 23 (FGF23) is a bone-derived hormone that plays a central role in the metabolic regulation of phosphate (P) and vitamin D[1–3]. FGF23 acts on the kidney to increase urinary P (U-P) excretion. The phosphaturic activity of FGF23 is based on its ability to suppress renal P reabsorption at the proximal tubules. Thus, FGF23 increases the P excretion at each nephron. Serum FGF23 levels are elevated in patients with chronic kidney disease (CKD). During CKD progression, FGF23 increases as early as at stage 2, long before serum P levels increase, which occurs at stage 4 or later. The progressive increase in FGF23 may be regarded as a response to the progressive decrease in the number of functional nephrons, which must be compensated by an increased P excretion per nephron to maintain the P homeostasis. Thus, in individuals with neutral P balance and equivalent P intake, a higher FGF23 suggests a lower number of nephrons. In fact, high serum FGF23 levels are associated with poor clinical outcomes in CKD patients [4–11].

Diabetes mellitus can cause CKD and is a risk factor for atherosclerosis and cardiovascular mortality. Although diabetic patients in early stages of CKD (stages 1 and 2) are reported to have lower FGF23 than nondiabetic patients matched for sex, age, and estimated glomerular filtration rate (eGFR)[12], FGF23 may still be useful as an early biomarker of CKD in diabetic patients. It was recently reported that a low fractional excretion of P (FEp)/FGF23 ratio is associated with severe abdominal aortic calcification in CKD stages 3 and 4 [13]. Because FGF23 suppresses P reabsorption at the proximal tubules, the fundamental role of FGF23 is to increase FEp. Thus, FEp/FGF23 is thought to represent the sensitivity of the kidney to FGF23.

In this report, we propose the ratio of U-P excretion (mg/day) to FGF23 as an index that theoretically represents the number of nephrons (nephron index)[1, 14]. Provided that FGF23 correlates with P excretion per nephron, namely,

$$\;P\;\text{excretion per nephron}=\;\frac{\text{Urinary P excretion}}{\text{Nephron number}}\;\propto \text{FGF}23$$

$$∴\text{Nephron number}\;\propto \;\frac{\text{Urinary P excretion}}{\text{FGF}23}\;\equiv \text{Nephron index}$$

If the Nephron index indeed reflects the functional nephron number, it will start decreasing in early stage CKD and potentially be useful for following up the progression, evaluating the risk for complications, and making a decision when to start the treatment in preemptive medicine. In the present retrospective, cross-sectional study, we determined whether the nephron index would be associated with renal function and vascular diseases in diabetic patients.

## Materials and Methods

### Subjects

The present study enrolled 142 subjects with type 2 diabetes mellitus. The cohort had a mean age of 63.0 years and included 73 men and 69 women. The subjects had been hospitalized at Jichi Medical University Saitama Medical Center to control their plasma glucose levels and complicated disorders. Blood samples after overnight fasting and daily total urine samples were collected from the subjects. Based on the KDIGO 2012 CKD guideline, the patients were classified into three groups according to eGFR degrees as follows: normal or high (G1; eGFR ≥ 90), mildly decreased (G2; eGFR 60–89), and mildly to moderately decreased (G3; eGFR 30–59). Subjects categorized as G4 (severely decreased, eGFR 15–29) were excluded from the present study. Thirty subjects had a history of treatment for macroangiopathy (30 had ischemic heart disease, 13 had cerebral vascular diseases, and 3 had peripheral arterial disease). Overall, 76 subjects had hypertension, 72 subjects had dyslipidemia, and 45 were smokers. None of the patients were administering vitamin D or calcium carbonate. The duration of the diabetes mellitus was estimated by the patients’ medical records. The occurrence of diabetic retinopathy was confirmed by an ophthalmologist.

### Measurements

Blood samples were collected into tubes and centrifuged at 3,000 rpm at 4°C for 15 min. The supernatants were decanted and frozen at −80°C until assayed.

Serum levels of HbA1c, Ca, P, 25OH vitD, FGF23, and intact PTH were determined. Serum FGF23 was measured by enzyme-linked immunosorbent assay (ELISA) using intact FGF23 ELISA kits (Kainos, Tokyo, Japan). The intra- and inter-assay coefficients of variation were <4.0% and 4%, respectively. Serum 25OH vitD was measured by ELISA using 25OH vitD ELISA kits (Immundiagnostik AG, Bensheim, Germany). The intra- and inter-assay coefficients of variation were both <7.0%. Serum FGF23 and serum 25OH vitD were measured in duplicate. Other variables were measured at a central laboratory section of the Jichi Medical University Saitama Medical Center. Renal function was determined by the eGFR, assessed by the Modification of Diet in Renal Disease equation revised for the Japanese population by the Japanese Society of Nephrology:

eGFR (ml/min/1.73 m^2^) = 194 × serum creatinine (mg/dl)^−1.094^ × age^−0.287^ × 0.739 (if female)[15]. The nephron index was calculated as the ratio of daily U-P excretion (mg/day) to serum FGF23 (pg/ml). The corrected serum Ca levels were calculated with the following equation: serum calcium + (4–serum albumin) (if serum albumin was ≤ 4 g/dl).

### Ethics Committee Approval

The study was approved by the Ethics Committee at Jichi Medical University Saitama Medical Center (No. 14–60) and performed in compliance with the Declaration of Helsinki. We obtained written consent from participants.

### Statistical Analysis

Data are expressed as mean ± standard deviation, and skewed variables are described as median with interquartile range. Clinical characteristics were compared among the three groups using one-way ANOVA or Kruskal-Wallis test. Two groups were compared by Student’s *t* test or Mann—Whitney *U* test, as appropriate. Categorical variables were compared by Fisher’s exact test. The *post-hoc* analyses was performed as needed (Holm test for serum FGF23 and Nephron index). Pearson’s correlation coefficient analysis was used for linear correlations between eGFR and serum P, intact PTH, FGF23, and Nephron index. Multivariate logistic regression analysis was conducted after univariate analysis. The variables were selected based on the comparison between with or without macroangiopathy. Namely, statistically significant variables were selected.

These variables were divided into two groups based on their averages in multivariate analysis. When a parameter revealed skewed distribution, the variable was transformed logarithmic parameters before analysis in correlation coefficient analysis and regression analysis. There was no missing data in each variable.

All analyses were performed with EZR (Saitama Medical Center, Jichi Medical University), a graphical user interface for R (The R Foundation for Statistical Computing, ver. 2.13.0) and a modified version of the R commander (ver. 1.6–3) that was designed to add statistical functions frequently used in biostatistics[16]. *p* < 0.05 was considered significant.

## Results

**Fig 1:**
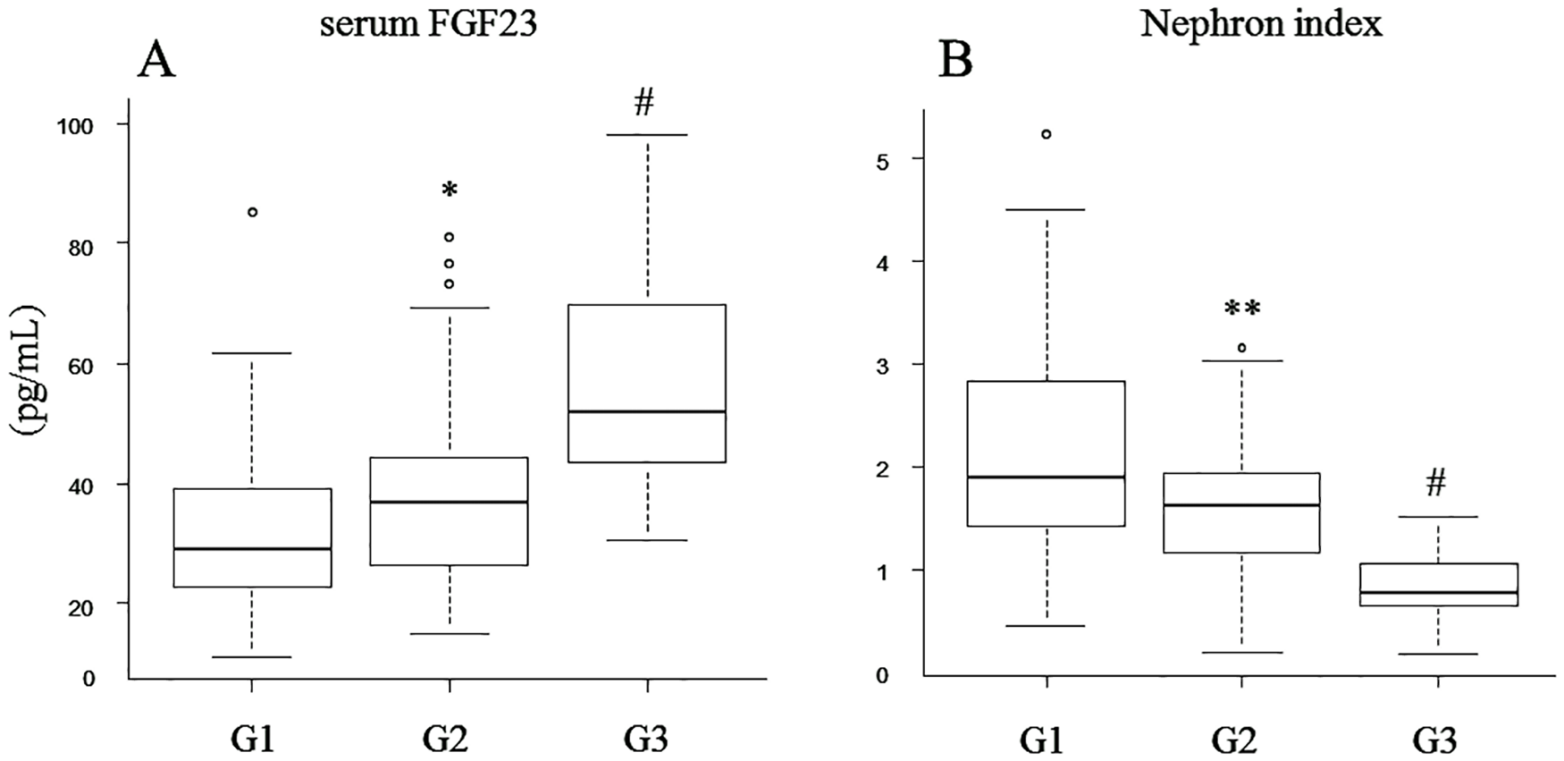
Comparisons of serum FGF23 (pg/mL). (**A**) and Nephron index (**B**) in chronic kidney disease stages G1, G2, and G3 in diabetic patients. *p < 0.05 vs. G1, **p < 0.01 vs. G1, #*p* < 0.01 vs. G2, *p* < 0.01 by post hoc analysis (Holm test) after Kruskal-Wallis test (*p* < 0.001).

**Fig 2:**
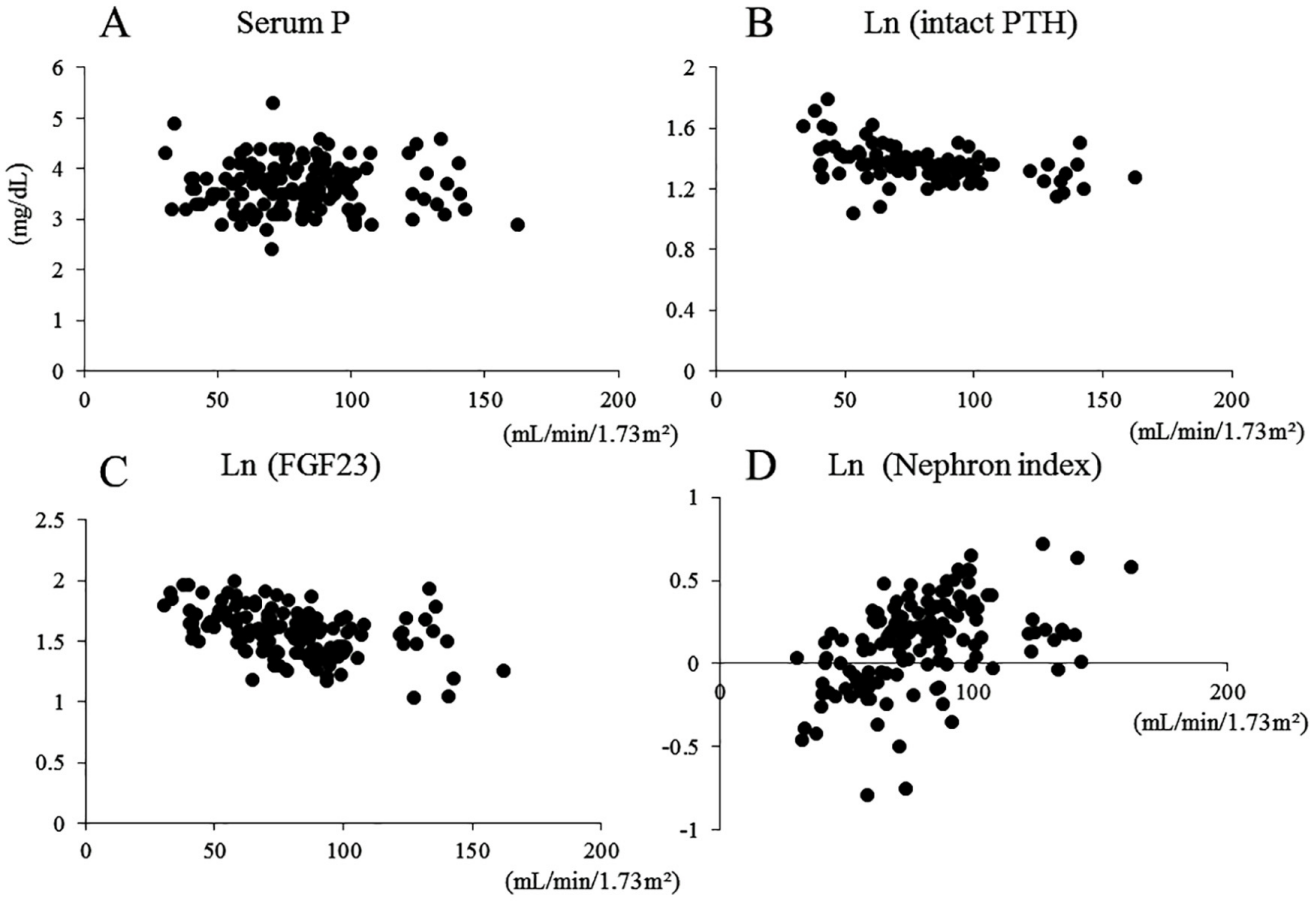
Simple regression analyses of serum P. (**A**), Ln intact PTH (**B**), Ln FGF23 (**C**), and Ln Nephron index (**D**) with eGFR in diabetic patients. Pearson’s correlation coefficients were calculated. FGF23, fibroblast growth factor 23; Ln, logarithmic value.

The clinical characteristics of the study participants are shown in Table 1. Considering all subjects, mean hemoglobin A1c (HbA1c) level was 9.3%, and the mean duration of diabetes mellitus was 12.1 years. Overall, 71 subjects had diabetic retinopathy. Considering all three CKD stage groups (G1: *n* = 40; G2: *n* = 70; G3: *n* = 32), the prevalence of hypertension, age, and duration of diabetes mellitus was significantly greater at the highest CKD stage class. There were no differences in serum calcium (Ca), P, and 25-hydroxyvitamin D3 (25OH vitD) levels. In contrast, serum FGF23 increased, and the nephron index decreased in association with the progression of CKD stage. A post-hoc analysis (Holm test) showed significant alterations in both FGF23 and nephron index initially at stage G2 (Fig 1). There was no difference in prevalence of diabetic microangiopathy (diabetic retinopathy and urinary albumin (U-albumin) levels). However, complicated macroangiopathy was significantly increased and associated with a higher CKD stage. A linear regression analysis model considering all the participants revealed a negative correlation of eGFR with logarithmic value log-transformed (Ln) intact parathyroid hormone (PTH) (*r* = −0.44, *p* < 0.001) and Ln FGF23 (*r* = −0.43, *p* < 0.001) and a positive correlation of eGFR with Ln nephron index (*r* = 0.47, *p* < 0.001), but not with serum P (*r* = −0.02, *p* = 0.78), as shown in Fig 2. Table 2 shows the difference in clinical parameters among subjects with (*n* = 30) and without (*n* = 112) macroangiopathy. The age and duration of diabetes mellitus were significantly higher in subjects with macroangiopathy. eGFR was significantly decreased in subjects with macroangiopathy compared with those without macroangiopathy. Regarding mineral metabolic parameters, U-P excretion and nephron index were significantly lower, whereas FGF23 was higher in subjects with macroangiopathy. There were no differences in serum Ca, P, intact PTH, and 25OH vitD levels. To determine the independent variables associated with the absence or presence of macroangiopathy, we performed a multiple logistic model analysis, shown in Table 3. Age, presence of hypertension, duration of diabetes mellitus, eGFR, serum FGF23, U-P, and nephron index were included in the model as independent factors, which parameters were significant variables in the comparison with or without macroangiopathy (based on Table 2). After univariate analysis of these seven variables, the final model showed that eGFR, duration of diabetes mellitus, and nephron index were independent factors for macroangiopathy.

**Table 1:** Clinical characteristics of the participants with CKD stages G1–G3. BMI, body mass index; DM, diabetes mellitus; SBP, systolic blood pressure; DBP, diastolic blood pressure; HbA1c, hemoglobin A1c; S-P, serum phosphate; U-P, 24-h urinary excretion of phosphate; S-Ca, serum calcium; 25OH vitD, 25-hydroxyvitamin D3; iPTH, intact parathyroid hormone; eGFR, estimated glomerular filtration rate; U-albumin, urinary albumin.

Variable	All individuals (142)	G1 (40)	G2 (70)	G3 (32)	*p* value
Age (years)	63.0 ± 11.0	54.5 ± 11.4	64.7 ± 9.1	69.8 ± 7.5	<0.001
Male sex (%)	51.4	55.0	52.9	43.8	0.639
Hypertension (*n*, %)	76 (53.5)	12 (30)	40 (57.1)	24 (75)	<0.001
Dyslipidemia (*n*, %)	72 (50.7)	16 (40)	41 (58.6)	15 (46.9)	0.233
Smoking (*n*, %)	45 (31.7)	7 (17.5)	28 (40)	10 (31.3)	0.052
BMI (kg/m^2^)	25.0 ± 4.4	24.1 ± 4.4	25.3 ± 4.8	25.3 ± 3.3	0.317
SBP (mmHg)	132 ± 19	129 ± 20	134 ± 18	134 ± 18	0.297
DBP (mmHg)	74 ± 12	75 ± 12	76 ± 10	69 ± 16	0.032
HbA1c (%)	9.3 ± 1.5	9.6 ± 1.5	9.3 ± 1.5	9.1 ± 1.6	0.372
Duration of DM (years)	12.1 ± 8.0	8.7 ± 7.2	12.2 ± 7.8	16.0 ± 7.8	<0.001
S-P (mg/dl)	3.6 ± 0.5	3.6 ± 0.5	3.7 ± 0.5	3.6 ± 0.4	0.829
U-P (mg/day)	539 ± 182	604 ± 204	551 ± 168	432 ± 132	<0.001
S-Ca (mg/dl)	9.2 ± 0.4	9.2 ± 1.4	9.3 ± 0.4	9.3 ± 0.48	0.712
25OH vitD (nmol/L)	81.3 ± 31.4	78 ± 29	84 ± 32	81 ± 34	0.721
iPTH (pg/mL)	23 [20–26]	21 [18–23]	23 [20–25]	27 [23–35]	0.001
eGFR (ml/min/1.73 m^2^)	79.5 ± 25.4	111 ± 19	76 ± 9	49 ± 9	<0.001
U-albumin (mg/day)	13.9 [6.6–47.5]	13.5 [4.9–26.2]	11.7 [5.6–37.9]	24.1 [10–113]	0.061
Retinopathy (*n*, %)	71 (50)	18 (45)	34 (48.6)	19 (59.4)	0.461
Macroangiopathy (*n*, %)	30 (21)	3 (7.5)	12 (17.1)	15 (46.9)	<0.001

**Table 2:** Differences in clinical parameters in subjects with (*n* = 30) and without (*n* = 112) macroangiopathy BMI, body mass index; DBP, diastolic blood pressure; DM, diabetes mellitus; eGFR, estimated glomerular filtration rate; FGF23, fibroblast growth factor 23; HbA1c, hemoglobin A1c; iPTH, intact parathyroid hormone; 25OH vitD, 25-hydroxyvitamin D3; SBP, systolic blood pressure; S-Ca, serum calcium; S-P, serum phosphate; U-albumin, urinary albumin; U-P, 24-h urinary excretion of phosphate.

Variable	Without	With	*p* value
Age (years)	61 ± 11	69 ± 9	<0.001
Male sex (%)	47.3	66.7	0.067
Hypertension (*n*, %)	54 (48.2)	22 (73.3)	0.022
Dyslipidemia (*n*, %)	54 (48.2)	18 (60)	0.311
Smoking (*n*, %)	35 (31.3)	10 (33.3)	0.828
BMI (kg/m^2^)	25.1 ± 4.7	24.6 ± 3.0	0.583
SBP (mmHg)	131 ± 18	137 ± 19	0.113
DBP (mmHg)	75 ± 13	71 ± 10	0.140
HbA1c (%)	9.4 ± 1.5	9.2 ± 1.6	0.504
Duration of DM (years)	10.8 ± 7.2	16.7 ± 9.3	<0.001
S-P (mg/dl)	3.6 ± 0.5	3.7 ± 0.5	0.725
U-P (mg/day)	564 ± 174	449 ± 183	0.002
S-Ca (mg/dl)	9.2 ± 0.4	9.2 ± 0.4	0.820
25OH vitD (nmol/L)	80 ± 34	85 ± 328	0.547
iPTH (pg/mL)	23 [20–26]	23 [20–27]	0.436
FGF23 (pg/mL)	37 [26–48]	44 [32–62]	0.020
Nephron index	1.58 [1.04–2.18]	1.05 [0.62–1.43]	<0.001
eGFR (ml/min/1.73 m^2^)	83 ± 25	65 ± 21	<0.001
U-albumin	14 [6.2–49]	13 [7.2–34]	0.908

**Table 3:** Multivariate logistic regression analysis showing independent variables in the assessment of patients with macroangiopathy. DM, diabetes mellitus; eGFR, estimated glomerular filtration rate; FGF23, fibroblast growth factor 23; U-P, 24-h urinary excretion of phosphate; OR, odds ratio.

Univariate Analysis				
Variable		OR	95% CI	*p* value
Age (years)	≥63	3.17	1.30–7.73	0.011
Hypertension	Present	2.95	1.21–7.19	0.017
Duration of DM (years)	≥12	3.11	1.31–7.40	0.010
eGFR (ml/min/1.73 m^2^)	<79	6.43	2.29–18.0	<0.001
Ln FGF23	≥1.57	2.07	0.89–4.82	0.091
U-P (mg/day)	≥540	2.39	1.03–5.57	0.043
Ln Nephron index	<0.13	3.60	1.54–8.44	0.032
Multivariate analysis				
Variable		OR	95% CI	*p* value
eGFR (ml/min/1.73 m^2^)	<79	3.63	1.19–11.1	0.024
Ln Nephron index	<0.13	2.80	1.01–7.74	0.047
Duration of DM (years)	≥12	2.71	1.07–6.89	0.036

## Discussion

When CKD progresses to stages 4 and 5, hyperphosphatemia induces phenotypic changes, transforming vascular smooth muscle cells into osteoblast-like cells, thus contributing to calcification of blood vessels. Elevation of serum FGF23 is associated with cardiovascular calcification and mortality in end-stage kidney disease[17–19]. In the present study, we demonstrated a close association of the nephron index but not of serum FGF23 with macroangiopathy in early-stage CKD. The nephron index decreased significantly compared with the elevation of serum FGF23 levels in early-stage CKD (G2) in diabetic patients. The index also had a positive correlation with eGFR and decreased in patients with macroangiopathy. In this retrospective study, patients with G1–G3 early-stage CKD were enrolled to reveal that the nephron index may reflect CKD progression or a decrease in the number of functional nephrons.

Several studies have reported a relationship between FEp and serum FGF23 levels in mild-to-moderate CKD stages. Dominguez *et al*. have revealed that higher serum FGF23 levels are more strongly associated with mortality and cardiovascular events when accompanied by lower FEp in a population with relatively mild CKD (mean eGFR 71 ml/min/1.73 m^2^)[20]. Beck *et al*. have reported that a high serum FGF23 level was a significant predictor of poor prognosis in subjects with CKD stage 3–4, but FEp did not modify the relationship between FGF23 and clinical outcome[21]. These studies suggested that a high FGF23 and low FEp reflected FGF23 resistance, yielding a poor phosphaturic response of FGF23 in the kidney. In our study, we focused on the nephron index, which could theoretically estimate the number of nephrons[1, 14]. Recently, Craver *et al*. showed that a low FEp/FGF23 ratio was associated with the severity of abdominal aortic calcification in patients with CKD stages 3–4 independent of age, gender, and presence carotid plaque[13]. Our pilot study showed a similar relationship between the nephron index and macroangiopathy in stage 1–3 CKD. In early-to-middle CKD stages, serum FGF23, but not serum P, is elevated. Serum FGF23 is one of the earliest markers of CKD, and an increase in FGF23 indicates excess P intake relative to the residual nephron number. In contrast, the nephron index theoretically reflects the number of functional nephrons and may be regarded as another early marker of early CKD stages. In multivariate analysis, identifying variables associated with macroangiopathy and the nephron index, but not FGF23, was an independent factor after adjustment for intact PTH and other variables. Several reports have pointed out the absence of a relationship between serum FGF23 and atherosclerotic disorders, for instance, arterial calcification[22], abnormal intima-media thickness[23], and peripheral artery disease[24]. In contrast, FGF23 has been associated with heart failure[7]. The clinical impact of serum FGF23 elevation in mineral and bone disorders (MBD) may be different among various stages of CKD. In our study populations, with a mean eGFR of 79.5 ml/min/1.73 m^2^, serum FGF23 was not an independent factor for macroangiopathy. Nakano *et al*. have shown that the serum FGF23 level in CKD stages 1 and 2 in diabetic patients is lower than that in nondiabetic patients due to dysfunction or decreased density of osteocytes and that diabetes mellitus affects vitamin D metabolism[12]. Indeed, in diabetic patients with osteoporosis, serum FGF23 level was decreased compared with those without osteoporosis. Moreover, serum FGF23 was related to the lumbar spine and femoral neck T-scores[23]. Therefore, there is a possibility that the clinical impact of serum FGF23 and MBD metabolic effects in a diabetic state may be different from those in a nondiabetic state. It is known that not only the glomerular damage but also the interstitial damage plays an important role in the progression of diabetic kidney disease[25]. The results of high FGF23 and low FEp may reflect diabetic tubular damage and loss of renal klotho. Therefore, it is necessary to evaluate the relationship between the nephron index and tubular markers, especially distal tubular damage markers since distal tubules express klotho.

There are limitations in our study. First, we could not determine whether the nephron index serves as an independent predictor of macroangiopathy in nondiabetic patients, such as in patients with CKD with primary glomerulonephritis, nephrosclerosis, and other kidney diseases. Second, we did not assess bone measures, including bone mineral density and medical history of bone fracture. It is known that patients with diabetes have abnormalities in bone metabolism (low bone formation) and an increased risk of hip and vertebral fractures[26–28]. The presence or absence of bone fractures or osteoporosis may have affected our results.

The present study demonstrates that the nephron index, which theoretically reflects the number of functional nephrons, is significantly decreased in early-stage CKD in diabetic patients. Furthermore, the number of nephrons is an independent risk factor for macroangiopathy. Further studies are needed to establish the nephron index as one of the earliest biomarkers of CKD.
